# Inhibitory Effects of Fruit Powders on the Formation of Polycyclic Aromatic Hydrocarbons in Charcoal-Grilled Pork

**DOI:** 10.3390/foods13193179

**Published:** 2024-10-06

**Authors:** Shang-Ming Huang, Bo-Chen Tung, Cheng-Hong Hsieh, Deng-Jye Yang, Ching-Wei Huang, Ling-Hsuan Chang, Kuo-Chiang Hsu

**Affiliations:** 1 Department of Nutrition, China Medical University, No. 100, Sec. 1, Jingmao Rd., Beitun Dist., Taichung City 40604, Taiwan; zxzxmj2323@mail.cmu.edu.tw (S.-M.H.); u112076007@cmu.edu.tw (B.-C.T.); u112211002@cmu.edu.tw (C.-W.H.); u111076006@cmu.edu.tw (L.-H.C.); 2 Department of Food Nutrition and Health Biotechnology, Asia University, 500 Lioufeng Rd., Wufeng, Taichung 41354, Taiwan; hyweuan@asia.edu.tw; 3 Institute of Food Safety and Health Risk Assessment, National Yang-Ming University, No. 155, Sec. 2, Linong St., Beitou Dist., Taipei City 11221, Taiwan; djyang@nycu.edu.tw

**Keywords:** PAHs, fruit powders, spraying, marinating, mixing

## Abstract

Polycyclic aromatic hydrocarbons (PAHs), carcinogenic substances primarily formed through pyrolysis and oxidation of fat at high cooking temperatures, are commonly found at high levels in grilled meats. Reducing PAHs formation by incorporating natural antioxidants, such as through marination, has been demonstrated to be effective. However, the inhibitory effect of fresh phenolic-rich fruit powders on PAHs formation in charcoal-grilled meats remains unknown. To clarify the application of the fruit powders, 15 experimental groups were conducted. All pretreatment techniques (spraying, marinating, and mixing) were applied across all four freeze-dried fruit powders (lemon, guava, papaya, and mango). Each method was systematically tested with each fruit powder to evaluate its effect on inhibiting the formation of the four PAHs (BaA, CHR, BbF, and BaP) in charcoal-grilled pork belly and loin. Firstly, guava powder exhibited the highest phenolic content and antioxidant activity compared to the lemon, papaya, and mango powders (*p* < 0.05), among which the main phenolic compounds were ellagic acid, quercetin, and epigallocatechin gallate (EGCG). Further, marination of pork belly with guava powder exhibited the highest inhibition rate of PAHs (94.8%), followed by lemon (91.1%), papaya (89.8%), and mango (89.0%), with a statistically significant difference at *p* < 0.05. The reduction in estimated daily intake (EDI) and the increase in the margin of exposure (MOE) indicate that consuming grilled meat treated with these fruit powders poses no safety concerns and may potentially reduce health risks. Finally, the sensory evaluation showed that marinating with guava powder did not perceptibly affect the sensory attributes of the meat. Overall, this study provides a potent strategy for inhibiting the formation of PAHs in meat during charcoal grilling by incorporating fruit powder while preserving sensory qualities.

## 1. Introduction

Polycyclic aromatic hydrocarbons (PAHs) are carcinogenic compounds produced during the combustion or high-temperature processing of organic materials. As endocrine disruptors, they exhibit carcinogenic, teratogenic, and mutagenic effects while also posing risks to the cardiovascular and respiratory systems [1,2]. More significantly, PAHs are a ubiquitous contaminant in food, especially in cooked meat [3,4], which typically arise from food-cooking procedures such as smoking, drying, roasting, and grilling [5]. The European Food Safety Authority (EFSA) has identified PAHs, including benz[a]anthracene (BaA), benzo[b]fluoranthene (BbF), chrysene (CHR), and benzo[a]pyrene (BaP), as appropriate indicators of the toxicity and occurrence of PAHs in food [6]. According to the panel, a sum of them would be a significant indicator of both the occurrence and toxicity of the genotoxic and carcinogenic PAHs compared to others alone [7]. Moreover, the European Commission, for example, has set limits at 2 μg/kg for BaP and 10 μg/kg for PAH4 (sum of BaA, CHr, BbF, and BaP) in oils and fats [7].

The primary approach to minimizing PAHs intake in food involves avoiding direct contact with flames during cooking [8]. However, the charcoal-grilled cooking method imparts a distinctive flavor and aroma to food, making it widely accepted and enjoyed by people. Therefore, finding effective ways to minimize PAH formation in meat during commonly used high-temperature cooking methods is essential. The mechanism behind its formation in charcoal-grilled cooking may be attributed to three main reasons [9,10]. These include the process of (1) thermal pyrolysis of organic matter (such as protein, carbohydrates, and lipids); (2) the dripping of fat or oil onto the heat source, resulting in the production of PAHs smoke, which adheres to the surface of the meat; and (3) incomplete combustion during heating, respectively. Thus, it is postulated that lipid is the primary component of PAHs formation and that lipid oxidation plays a significant role in the formation and accumulation of PAHs during meat processing [11].

In recent years, natural products have been found to have significant inhibitory effects on forming PAHs, such as meat seasoning with complex mixtures of spices, beverages, and plant extracts [12]. Lipid oxidation is a chain reaction involving free radicals during the formation of PAHs. A considerable body of evidence indicates that the inhibitory mechanisms against PAHs formation during the cooking process have been attributed to the free-radical scavenging activity and the presence of phenolic compounds in these edible materials [13,14,15,16]. Antioxidants, particularly those with phenolic structures, can scavenge free radicals and reactive oxygen species (ROS) generated during high-temperature cooking, such as grilling or smoking. By neutralizing these reactive species, antioxidants can prevent the oxidative degradation of fats, a process that leads to the formation of PAHs [17]. For example, marinating chicken wings with green, white, and yellow tea, known for their high phenolic content, before charcoal grilling led to reductions in PAHs levels of 57%, 31%, and 23%, respectively. The phenolic compounds in the tea infusion adhered to the chicken wing surface, scavenging free radicals generated during the grilling process and thus inhibiting the formation of PAHs [15]. Onion and garlic contain various flavonoids, including chlorogenic acid and catechin, which can inhibit the formation of PAHs when added to meat mixtures [18].

Fruits are a rich source of antioxidants such as vitamins C and E, polyphenols, and carotenoids [19]. It has been demonstrated that antioxidants can inhibit the formation of PAHs [20], rendering fruits as a promising option. Nevertheless, to date, only a limited number of studies have investigated the impact of the constituents present in fruit on the formation of PAHs [21]. Compared to fruit juice, fruit pulp contains a greater concentration of phenolic compounds and demonstrates superior antioxidant capabilities. For example, whole lemon fruit contains a significantly higher concentration of phenolic compounds than lemon juice [22], and fruit pulp exhibits a greater DPPH free radical scavenging activity than fruit juice [23]. Furthermore, the freeze-drying method was identified as the most effective method for preserving phenolic compounds, as evidenced by the highest total phenolic content observed in mango powder compared to other drying methods [24].

As of now, the influence of incorporating fruit powders on PAHs formation in cooked meat has not been extensively investigated. In this study, four kinds of fresh fruits native to Taiwan, including lemon, guava, papaya, and mango, were selected to produce fruit powder by freeze-drying. The efficacy of three powder-addition methods, including spraying, marinating, and mixing, was then compared in charcoal-grilled pork. The effects of fruit powders on different types of meat, including pork belly and loin, were also investigated to evaluate whether the fat content influences the formation of PAHs. Furthermore, the risk assessment and the sensory evaluation were conducted to gain further insight into the impact of fruit powders on the safety and acceptability of meat products. This study provides a theoretical foundation for utilizing natural fruit powders as a mitigation strategy to lower the risk of PAHs in grilled meats while preserving their sensory properties.

## 2. Materials and Methods

### 2.1. Fruit Powders Sample Preparation

The fruits, namely lemon (*Citrus lemon*), guava (*Psidium guajava*), papaya (*Carica papaya*), and mango (*Mangifera indica*), were purchased from a local supermarket (Taichung, Taiwan). All fruits were gently washed with tap water. The seeds of the peeled papaya and mango, as well as skinned lemon and guava, were removed. The fruit pulp was cut into 1 cm chunks and freeze-dried for 72 h. The freeze-dried fruit pulp was then ground with a grinder and passed through a 40-mesh screen to collect the powders of a similar size. Each fruit powder was vacuum-sealed separately in a polyethylene bag and stored in a dark, temperature-controlled environment at room temperature for a minimum of 30 days prior to use. In this study, all subsequent tests on four kinds of fruits were performed in independent batches.

### 2.2. Reagents

The DPPH reagent (2,2-diphenyl-1-picrylhydrazyl) and ferric chloride were purchased from Sigma-Aldrich Co. (St. Louis, MO, USA). Ethanol, potassium ferricyanide, and trichloroacetic acid were purchased from J.T. Baker (Philipsburg, NJ, USA). Acetonitrile, acetone, and methanol were purchased from Merck Co. (Darmstadt, Germany). The standards of the EU priority PAHs (purity), including BaA (99%), BaP (99%), BbF (99%), and CHR (99%), were obtained from Restek Co. (Bellefonte, PA, USA). The QuEChERS kits were obtained from Dikma Technologies Co. (Dikma Technologies Inc., Lake Forest, CA, USA).

### 2.3. DPPH Scavenging Activity

The fruit powders were dissolved in 10 mL of 70% ethanol in each diluted concentration and vortexed for 2 min. The tubes were subjected to an ultrasonic bath for 40 min at room temperature and then centrifuged at 4 °C 3000 rpm for 10 min. The resulting supernatants were then collected. The DPPH radical scavenging assay was performed following the procedure of Akharaiyi et al. [25], with some modifications. Each sample was added to a tube containing 0.5 mM DPPH (dissolved in 70% ethanol) at a ratio of 1:9. Following a 30 min incubation period in the dark, the absorbance at 517 nm was measured. The radical scavenging activity (RSA) was calculated using the formula below, demonstrating the concentration for 50% of the maximal effect (EC_50_).

$$RSA\%=\left(1-\frac{A_{Sample}}{A_{Control}}\right)\times 100\%$$

where A is the absorbance at 517 nm.

### 2.4. Total Phenolic Content (TPC)

First, 2 g of each fruit powder was dissolved in 20 mL of 70% ethanol. The tube was vortexed for 2 min and shaken for 30 min. The tube was centrifuged at 4 °C and 3000 rpm for 15 min to collect the supernatant, after which the above procedure was repeated thrice. The supernatant was concentrated at 40 °C for 40 min and quantified to 25 mL to obtain the fruit powder extract. The total phenolic content of fruit powders was determined using the Folin–Ciocalteu method [26]. A total of 250 μL of each fruit powder extract/standard was taken, followed by the addition of 250 μL of 1N Folin–Ciocalteu reagent and 3 mL of distilled water. The mixture was vortexed and incubated at room temperature in the dark for 8 min. Subsequently, 750 μL of 20% Na_2_CO_3_ and 950 μL of distilled water were added to the mixture and then incubated at room temperature in the dark for a further 30 min. The absorbance at 765 nm was then measured, and the results were expressed in mg of gallic acid equivalent per 100 g of dry powder.

### 2.5. Phenolic Compound Analysis

Each fruit powder sample (0.5 g) was extracted with 10 mL of methanol (95%) and shaken for 60 min at room temperature in a tube. The tube was centrifuged at 3000 rpm at room temperature for 10 min, after which the supernatant was collected. The precipitate was extracted twice with 10 mL and 5 mL methanol, respectively, and quantified to a total volume of 25 mL. The supernatant was then filtered through a 0.45 μm filter for further analysis.

Samples and phenolic compound standards were analyzed by an ACQUITY™ UPLC system (Waters, Milford, MA, USA) coupled with an AB Sciex 6500 quadrupole ion trap mass spectrometer (AB Sciex, Framingham, MA, USA) with an electrospray ionization (ESI) source. The chromatographic separation was conducted on an Atlantis T3 column (2.1 × 150 mm, 3 μm) maintained at 35 °C using a binary mobile phase. The mobile phase A consisted of 0.1% formic acid in the water, while the mobile phase B was 0.1% formic acid in acetonitrile. The injection volume was set at 5 μL. The flow rate employed was 0.25 mL/min. The gradient elution program was as follows: 0–1 min by 1% B; 1–15 min by 1–40% B; 15–18 min by 40–99% B; 18–21 min by 99% B; 21–21.5 min by 99–1% B; 21.5–25 min by 1% B, respectively. Chromatograms and mass spectral data were acquired and processed using SCIEX OS 2.0 software (AB Sciex, Framingham, MA, USA).

### 2.6. Treatments of Fruit Powder on Charcoal-Grilled Pork Sample

The pork belly and loin were purchased from a local supermarket (Taichung, Taiwan). The meats were then cut into approximately 1 cm thick slices (each piece weighing approximately 50 g, with a length of 6 cm and a width of 5 cm). Three distinct methods were employed to incorporate fruit powders into the meat, including spraying, marinating, and mixing. In this study, each piece of meat was individually treated through spraying, marinating, and mixing methods. Additionally, three replicates were conducted using independent batches of fruit powder and meat pieces throughout the experiments. A 1.2% fruit powder solution was prepared for the spray and marinade methods. The spray method used 3 g solution on both sides of the meat pieces before being charcoal-grilled. In the marinade method, the meat pieces were prepared by marinating them in a 1.2% fruit powder solution (1:1 *w*/*w*) for 20 min prior to the cooking process. In the mixed method, meat pieces were ground to make meat patties, and 0.6 g of fruit powder was added and kneaded evenly into round patties in the mixing bowl. Once all flames had been wholly ignited, the meat was cooked thoroughly for around 10 min, with frequent turning. All samples were stored at −20 °C until analysis.

### 2.7. Analysis of PAHs

The PAHs analysis was conducted according to the QuEChERS method of Chiang et al. [27], with slight modifications. A ground sample (2 g) was placed in a tube and mixed with 10 mL of deionized water and a ceramic stone. The tube was vortexed for 1 min. Subsequently, 10 mL of acetone was added and vortexed for 1 min. Subsequently, the contents of the QuEChERS column (comprising 4 g of MgSO_4_ and 1 g of sodium acetate) were added to the tube and shaken for 1 min. The tube was then centrifuged at 4000 rpm for 5 min. For purification, the supernatant was transferred into a QuEChERS clean-up column (comprising 900 mg of MgSO_4_, 300 mg of primary secondary amine (PSA), and 300 mg of end-capped octadecylsilane silica gel particles). The QuEChERS clean-up column was shaken for 1 min and then centrifuged at 4000 rpm for 5 min. The supernatant was subsequently collected for further analysis of PAHs.

The HPLC system includes an LC-10AT HPLC pump system, an SCL10A controller, a SIL-10A auto-sampler, an RF-10AxL FLD (Shimadzu Co., Kyoto, Japan), and an S-3210 photodiode-array (PDA) detector (Schambeck SFD GmbH, Bad Honnef, Germany). The PAHs were analyzed under the following conditions: A Pinnacle II PAH column (150 mm × 3.0 mm ID, 4 μm) (Restek Co., Bellefonte, PA, USA) was kept at 35 °C. The mobile phase A comprised double-distilled H_2_O, and mobile phase B was acetonitrile using 4% of THF. The gradient elution proceeded as follows: 0–3 min by 70% B; 3–15 min by 70–90% B; 15–15.5 min by 90–100% B; finally, the column was maintained at 100% B for the final 5 min. The flow rate was initially set at 1.4 mL/min for the first 15 min, then increased to 1.4–2.0 mL/min for the subsequent 5 min, and then increased to 2.0 mL/min for the final 5 min. The injection volume was set at 20 µL.

### 2.8. Risk Assessment 

In this study, the Nutrition and Health Survey in Taiwan (NAHSIT) database acquired from the Taiwan Ministry of Health and Welfare was subjected to analysis. The database encompassed all age groups (1–65 years old), excluding special ethnic groups. The risk assessment focused on the pork belly and the pork loin intake required for the study, as detailed in Chiang et al. [28]. The database indicates that the average daily intake (ADI) of pork belly is 0.14 g/kg body weight/day. The ADI of pork loin is 0.04 g/kg body weight/day.

The margin of exposure (MOE) was employed as a qualitative risk indicator to evaluate the presence of carcinogens in food [29]. MOE can only represent the degree of concern but not quantify the risk. Genotoxic and carcinogenic substances are quantified by the ratio of the dose of a substance that causes adverse reactions in animals to the human dietary intake. The MOE was evaluated as follows:

$$MOE=\frac{{BMDL}_{10}}{EDI}=\frac{{BMDL}_{10}}{\sum_{k=1}^{n}\left[C_{k,j}\times {\left(\frac{{CR}_{k}}{BW}\right)}_{i}\right]}$$

The benchmark dose level (BMDL_10_) is a study endpoint associated with a 10% increase in the risk of adverse effects in the exposed test animals compared to the background levels of risk. The unit of measurement is mg/kg body weight/day. The estimated daily intake (EDI) is defined as the presumed daily exposure to or consumption of a nutrient or chemical residue. In the formula, *n* is the amount of food *k* ingested by individual *i*; C*_k,j_* is the average concentration of food hazard *j* in the *k* category food sample; CR*_k_* is the food intake (g/day) of food *k* by individual *i*; BW is the body weight (kg) of individual *i*.

The BMDL_10_ used in this study was first announced by the European Food Safety Authority (EFSA). The BMDL_10_ of BaP is 0.07 mg/kg body weight/day, and the BMDL_10_ of PAH4 is 0.34 mg/kg body weight/day. To classify the risk interpretation of MOE values, the following bandings were employed: MOE values > 1,000,000 are considered a negligible concern, while values between 100,000 and 1,000,000 are classified as a “negligible concern with action minimizing future exposure. Values between 10,000 and 1,000,000 are categorized as a low concern, while those <10,000 are regarded as a possible concern [30]. This indicates that the MOE is on the rise, accompanied by a decline in the level of concern [31].

### 2.9. Sensory Evaluation of Meat

To ensure that the sensory panelists could accurately differentiate between the samples, we provided them with specific training tailored to this study. Most of the panelists had prior experience in sensory evaluation and regularly participated in similar studies and were thus familiar with primary sensory evaluation methodologies. However, for this particular experiment, we conducted additional training to ensure they understood how the freeze-dried fruit powders (lemon, guava, papaya, and mango) could affect the flavor, texture, and appearance of the charcoal-grilled pork.

During the training sessions, panelists participated in a series of preliminary evaluations designed to familiarize them with the sensory evaluation forms and the product’s specific attributes, such as appearance, smell, texture, flavor, greasiness, and overall acceptance. We also emphasized correctly distinguishing the subtle differences between samples treated with different fruit powders, and group discussions were conducted to align the panelists on sensory terminology. These targeted training sessions ensured that each panelist was fully equipped to understand and differentiate between the samples, resulting in more accurate and reliable sensory evaluation data.

In this study, the sensory evaluation of charcoal-grilled pork belly was conducted using two distinct sensory models: the triangle test and the 7-point scale scoring difference test. A single sensory evaluation session was conducted (Supplementary Materials), in which all panelists tested samples from all treatment conditions, including both the marinated and non-marinated pork belly. The same ten panelists participated throughout the session, ensuring consistency in the evaluation. Since only one session was conducted, all samples were tested during this single session. Informed consent was obtained from all panelists prior to their participation in the sensory testing, in accordance with ethical standards for research involving human subjects, and all procedures were conducted in compliance with the relevant guidelines for sensory evaluation.

For the triangle test, the procedure followed the method described by Lawless and Heymann [32]. During a single sensory evaluation session, ten trained panelists participated. Each panelist was presented with three blind samples, two identical and one different, each labeled with randomly generated numbers. The panelists were asked to identify the sample that differed from the others. Samples tested included both pork belly marinated with a guava powder solution and those without the marinade. The significance of the triangle test results was determined using the minimum number of correct responses required for statistical validity, following ISO 4120:2016 guidelines. This method ensures the robustness of the results, and samples that did not show significant differences were marked with the same letter (*p* > 0.05), indicating sensory similarity.

In addition to the triangle test, the degree of preference for the pork belly was assessed using a 7-point scale scoring difference test (Supplementary Materials). The panelists rated the pork on a scale from 1 to 7, where 1 represented “dislike extremely”, and 7 represented “like extremely”. Intermediate scores were defined as follows: a score of 2 indicated “dislike moderately”, 3 was “dislike slightly”, 4 denoted “neither like nor dislike”, 5 reflected “like slightly”, and 6 indicated “like moderately”. The sensory attributes evaluated included appearance, smell, texture, flavor, greasy level, and overall acceptance. The scores reflected the panelists’ personal preferences for each sensory characteristic.

### 2.10. Statistical Analysis

In this study, two statistical models were employed to analyze the sensory data and the overall experimental data analysis. For the triangle test, the significance was evaluated based on the minimum number of correct responses required, as specified by ISO 4120:2016. This standard defines the threshold for statistical significance in difference testing, ensuring that the results are not due to chance. For the 7-point scale scoring difference test, a one-way ANOVA was used to analyze the sensory data. This model accounted for the fixed effects of treatment groups (e.g., control and experimental groups with different treatments) and panelists as well as the interaction between these effects. Although only a single sensory evaluation session was conducted in this study, the model structure typically accounts for variance due to sensory sessions when applicable. However, no session-related variance was included in this case, as there was only one session. Instead, the random effects considered were limited to individual panelist preferences. The data from the experiments, including the DPPH radical scavenging activity (EC_50_) and total phenol content (TPC) of four different fruit powders, as well as their impact on polycyclic aromatic hydrocarbons (PAHs) in charcoal-grilled pork after various treatments were analyzed using one-way ANOVA. Statistical analysis was conducted with SAS software (version 9.4; SAS Institute, Cary, NC, USA). The Bonferroni post hoc test was used to identify significant differences among the mean values, with statistical significance set at *p* < 0.05.

## 3. Results and Discussion

### 3.1. DPPH Antioxidant Activity, Total Phenolic Content, and Phenolic Compounds of Fruit Powders

As shown in Table 1, the highest antioxidant activity in scavenging DPPH radicals was exhibited by guava powder, with an EC_50_ value of 2.97 mg/mL, followed by mango powder (6.42 mg/mL), lemon powder (7.73 mg/mL), and papaya powder (8.26 mg/mL), respectively. Furthermore, the highest content of phenolics was also found in guava powder (469.6 mg GAE/100 g), followed by lemon (367.5 mg GAE/100 g), papaya (325.2 mg GAE/100 g), and mango (367.5 mg GAE/100 g), respectively. Compared to other fruits, guava powder exhibited potential antioxidant properties, as evidenced by its lower EC_50_ value of DPPH and higher total phenolic content, findings consistent with those reported in previous studies on fruits [33,34]. For example, the antioxidant activity of 36 different fruits from Taiwan was assessed, with the guava found to rank second to the mulberry. Additionally, the soluble free phenolic compounds content was higher than the papaya and mango [33]. In another study involving 27 kinds of fruit in Singapore, it was observed that guava exhibited the highest antioxidant activity (270 mg L-ascorbic acid equivalent/100 g), surpassing that of mango, lemon, and papaya [34].

The antioxidant activity of natural products is commonly found to be strongly associated with their phenolic composition [35]. In this study, the phenolic compounds in four different fruit powders were also determined by UPLC. As shown in Table 2, guava powder exhibited the highest content of EGCG (0.36 µg/g), followed by mango (0.10 µg/g) and papaya (0.04 µg/g) powder. The highest content of ellagic acid was observed in guava powder (29.67 µg/g), followed by mango (16.82 µg/g), papaya (9.39 µg/g), and lemon (3.41 µg/g), while the highest content of quercetin was observed in lemon powder (5.41 µg/g). Gallic acid was only identified in papaya (0.88 µg/g) and mango (2.02 µg/g). It is in agreement with this that the ellagic acid content of guava is notably higher than that of the other 25 kinds of fruits [36]. Additionally, gallic acid is also one of the most abundant phenolic compounds in mango [37].

It can be speculated that the antioxidant activity exhibited by EGCG, ellagic acid, quercetin, and gallic acid may be more affected by their respective concentrations, as evidenced by EC_50_ values when evaluated independently, indicating that EGCG is the most active (3.6 µM), followed by ellagic acid (<5 µM), quercetin (3.3–5.5 µM), and gallic acid (2.5–10.1 µM) [38,39,40,41,42]. The findings of this study indicate that the presence of higher levels of EGCG and ellagic acid in guava and mango powder may contribute to enhanced antioxidant activity. On the other hand, antioxidant activity may also be related to the number of functional groups present. EGCG contains the most hydroxyl groups (eight), followed by quercetin (five), ellagic acid (four), and gallic acid (three), while the latter group each contains two or three hydroxyl groups. Following the structure–activity relationship theory, an increased number of hydroxyl groups is posited to result in heightened activity in the scavenging of free radicals, chelation of metal ions, and the transfer of hydrogen atoms or electrons [43,44]. Consequently, fruits containing a certain amount of phenolic compounds, especially EGCG, quercetin, ellagic acid, and gallic acid, may offer more significant antioxidant potential. Herein, our results indicated that guava powder exhibits the highest DPPH antioxidant activity, potentially attributed to its possession of the highest amount of phenolic compounds mentioned above, which may have the potential to inhibit the formation of PAHs in roasted meats.

### 3.2. Inhibitory Effect of Fruit Powders on PAHs Formation in Charcoal-Grilled Pork

Table 3 shows the levels and inhibition rates of PAHs in charcoal-grilled pork after different treatments of fruit powders. The results showed that after charcoal grilling, BaA, CHR, BbF, BaP, and PAH4 contents were higher in pork belly than in pork loin. According to the TFDA database, the fat content of pork belly is 32.9%, whereas pork loin is 14.4%. Previous studies have shown that higher fat content in meat leads to higher PAHs production [45,46].

Using fruit powers resulted in higher PAH4 inhibition rates in pork belly (11.5–94.8%) than in pork loin (12.7–85.0%). Inhibition rates were also higher for marinade treatments (47.7–94.8%) than spray treatments (11.5–65.7%). However, guava powder showed the highest PAH4 inhibition in both spray and marinade treatments for pork belly, reducing PAH4 from 87.28 µg/kg to 29.97 µg/kg (65.7% less than the control) in the spray treatment and from 43.74 µg/kg to 2.33 µg/kg (94.8% less than the control) in the marinade treatment. The inhibition rates of other fruit powders for pork belly were, in decreasing order, lemon (55.9%), papaya (48.4%), and mango (11.5%) for spray treatments and lemon (91.1%), papaya (89.8%), and mango (89.0%) for marinade treatments.

In pork loin, lemon powder significantly reduced the PAH4 (30% reduction compared to the control), followed by guava, mango, and papaya. Papaya powder also significantly reduced PAH4 (63% less than the control), with subsequent inhibition rates observed for mango, guava, and lemon. When mixing treatments were used, fruit powders demonstrated a potent inhibitory effect on PAHs (over 78.3% inhibition in the belly and over 79.4% inhibition in the loin), with guava powder showing the highest inhibition rate in pork belly (90.4%). In particular, the controls in the mixing group exhibited the highest levels of PAH4 compared to the other treatments. A previous study indicated that PAH4 levels in pork belly can reach over 300 µg/kg [47]. The homogenization of the meat by whipping under mixing conditions exposed the tissue structure of the meat, which in turn led to the scattering and covering of oil molecules over the surface, thus facilitating the formation of PAHs. Therefore, it was observed that the PAHs produced in the mixing process were relatively higher in concentration. In addition, it was also found that the inhibition rates for mixed treatments in both belly and loin were similar, while spray and marinade treatments showed higher inhibition rates in pork belly. Currently, among the three different applications of fruit powders, the marinade method shows the most significant inhibitory impact on the formation of PAHs.

Due to their lipophilic and hydrophobic properties, PAHs accumulate in the food chain and are commonly generated during high-temperature cooking processes, such as grilling [48]. Among the various sources of dietary exposure to PAHs, oil is considered a primary one. Although the precise mechanism of PAHs formation in oil remains uncertain, some researchers have postulated that PAHs formation is strongly correlated with fats, suggesting that numerous free radicals may be generated during the oxidation of fatty acids to hydroperoxides, which subsequently form PAHs through small molecules polymerization or intramolecular addition. During this process, numerous studies have demonstrated that the formation of PAHs can be inhibited by phenolic compounds due to their antioxidant properties [49]. For example, the highest inhibition rate of PAHs (82%) when sprayed on charcoal-grilled pork loin was observed with elderberry vinegar, which may be attributed to its high levels of cyanidin-3-sambubiose and cyanidin-glucoside [16]. PAHs produced from charcoal-grilled chicken wings can be inhibited by green tea (57% inhibition rate), which contains phenolic compounds (-)-epigallocatechin-3-O-gallate, quinic acid, and gallic acid [15]. Other studies also demonstrated that eight kinds of pure phenolic compounds (including EGCG, GC, C, ECG, CG, eriodictyol, naringenin, and quinic acid) and the phenolic compounds present in beer (including p-coumaric acid, ferulic acid, and catechin) have an inhibitory effect on PAHs when marinating charcoal-grilled chicken wings [14,20]. It is apparent that phenolic compounds interact with fat, thereby inhibiting the progression of fat oxidation and stabilizing the structure of proteins through covalent bond interactions, which is facilitated by an increased number of hydroxyl (OH) groups on the molecule, allowing for a greater degree of protein stability [50].

Currently, four kinds of fruit powders have been demonstrated to effectively inhibit the formation of PAHs, which may be attributed to the antioxidant activity of the main phenolic compounds (EGCG, ellagic acid, quercetin, and gallic acid) present in fruit powders (Table 2). Among them, guava powder has the highest EGCG and ellagic acid content. Moreover, marinating demonstrated a more significant inhibitory effect than the spraying and mixing methods. One possible explanation for this result is that the meat was pre-soaked in a solution containing fruit powder, which resulted in the fruit ingredients, including phenolic compounds, entering the meat tissue in advance as a free radical inhibitor. Furthermore, marinade can act as an effective barrier, impeding direct contact between the meat and the heat source. In the marinade method, guava powder exhibited the highest inhibition rate of 94.8% on PAHs, with its rich composition and content of phenolic compounds serving as the primary reason for this inhibitory effect.

### 3.3. Risk Assessment of PAHs

Risk assessment terms like EDI and MOE quantify health risks by estimating daily intake of harmful substances (EDI), where a reduction indicates lower exposure, and evaluating the safety margin between harmful and actual exposure levels (MOE), where an increase suggests reduced health risks. The EDI and MOE for the general population are shown in Table 4. The risk assessment was conducted to examine the effects of different treatments of fruit powders on charcoal-grilled pork belly and loin. The results indicated that the EDI was lower, while the MOE was higher for both BaP and PAH4 when using fruit powders. Additionally, the risk associated with consuming pork belly was found to be higher than that of consuming pork loin. The two primary reasons for this phenomenon can be identified as follows: Firstly, in Taiwan, people consume more belly than loin meat. Secondly, the consumption of belly meat produces higher levels of PAHs than that of loin meat. Our results further indicated that marinating pork belly with guava powder can significantly reduce the BaP EDI from 1.77 to 0.11 ng/kg bw/day. Other fruit powders, such as lemon, papaya, and mango, reduced the BaP EDI to 0.1–0.15 ng/kg bw/day. The MOE values also demonstrated that all the fruit powders increased the MOE, indicating a decreased risk of PAHs.

The mixing control group exhibited the highest EDI at 12.91 ng/kg bw/day, with a MOE below 10,000, indicating a significant health risk. However, when guava powder was applied to the pork belly, the EDI was reduced to 0.92 ng/kg bw/day, and the MOE reflected a low health risk. Furthermore, marinating the pork belly increased the MOE from 39,000 to over 100,000, indicating a negligible concern. For the loin, the EDI in the mixing group decreased from 0.31 to 0.0–0.07, with the MOE showing only a marginal effect.

The results of the PAHs evaluation indicated that the EDI was higher in the control group than in the fruit powders group. Moreover, the EDI for pork belly was found to be higher than that for pork loin. Mixing the belly with guava powder could reduce the EDI to 3.44 ng/kg bw per day. Furthermore, the MOE also exceeded the threshold for marginal effect. Consequently, fruit powders significantly reduced the risk associated with charcoal-grilled pork, especially pork belly.

In summary, this study found that four kinds of fruit powders effectively reduce EDI and significantly lower the risk of MOE, regardless of how they are used. However, the risk associated with consuming belly is indeed higher than with loin due to the higher oil content in belly meat, which leads to producing more PAHs. Additionally, the overall risk assessment for belly meat is elevated due to the higher food intake. Consequently, for people aiming to eat healthier and safer, it is recommended to choose loin cuts over belly cuts, as they present a lower risk of PAHs.

### 3.4. Food Sensory Evaluation

Sensory evaluation is crucial in assessing the success of any PAH-inhibiting treatment because food quality is not solely judged by its safety but also by its sensory attributes, including appearance, smell, flavor, greasiness, and overall acceptance. While reducing PAH formation in grilled or smoked meats is vital for health, such treatments must not compromise the eating experience. In this study, the sensory evaluation of charcoal-grilled pork belly was conducted using two distinct sensory models: the triangle test and the 7-point scale scoring difference test.

In the triangle test involving 10 panelists, at least 7 (>70%) were required to correctly identify the difference between the samples for the result to be considered significant. This experiment aimed to test the hypothesis that adding fruit powder to marinated meat before charcoal grilling would reduce the formation of PAHs without significantly affecting the original flavor of the meat. As shown in Table 5, only two panelists (20%) could distinguish between the samples. It can be inferred that marinating pork belly with a guava powder prior to charcoal grilling did not significantly alter the flavor of the meat. This outcome aligns with the research objective of ensuring whether adding fruit powder would affect the original flavor of the meat.

Additionally, the average scores on the sensory evaluation analysis showed no statistically significant difference between the control and experimental groups across five indicators, including appearance, smell, flavor, greasiness, and overall acceptance (Table 6). Therefore, it can be concluded that marinating with guava powder did not significantly influence the sensory qualities of the food compared to the control. Herein, our research provides the concept of using fruit powder to mitigate the formation of PAHs in charcoal-grilled pork without compromising the original taste of the meat.

## 4. Conclusions

This study demonstrates for the first time the potential of fruit powders, abundant in phenolic compounds, as effective agents in inhibiting the formation of PAHs when employed in charcoal-grilled pork belly and loin. Notably, the findings indicate that pork belly marinated with 1.2% guava powder exhibited the highest inhibitory effect on the formation of PAHs. This study provides a theoretical basis for utilizing natural sources, such as fruit powders, as inhibitors of PAHs during the charcoal grilling of meats. This approach might provide a practical solution to significantly lower the risk of PAHs exposure from dietary sources without affecting the sensory qualities of the food.

Future research on PAH inhibition in grilled meats should focus on several key areas. Expanding studies to include different types of meat (e.g., beef, chicken, and fish) will help determine whether the PAH-inhibiting effects of fruit powders are consistent across various fat compositions and grilling properties. Larger-scale trials with diverse meat samples and conditions would increase the reliability and applicability of findings, ensuring reproducibility in commercial settings. Additionally, comprehensive sensory evaluations are necessary to confirm that PAH-reducing treatments do not adversely impact flavor, texture, or consumer acceptability. Further optimization of fruit powder treatments, including refining concentration, marination duration, spray intensity, and powder combinations, will maximize PAH inhibition while preserving meat quality. Lastly, long-term health studies should explore the broader implications of reduced dietary PAH exposure and the potential health benefits of using natural antioxidants in routine food preparation.

## Figures and Tables

**Table 1:** DPPH radical scavenging activity (EC_50_) and total phenol content (TPC) of four kinds of fruit powders.

Fruit Powder	DPPH EC_50_ (mg/mL) *	TPC (mg GAE/100 g Dry Powder)
Guava	2.97	469.6 ± 2.27 ^a^
Lemon	7.73	367.5 ± 11.42 ^b^
Papaya	8.26	325.2 ± 3.69 ^c^
Mango	6.42	288.4 ± 4.60 ^d^

* Sample concentration for 50% removal of DPPH radicals. Total phenol content is shown as the mean ± SEM (*n* = 3). Significant differences between the samples are expressed as different letters (*p* < 0.05).

**Table 2:** Content of phenolic compounds in different fruit powders (µg/g dry powder).

Phenolic Compound	Guava	Lemon	Papaya	Mango
EGCG	0.36 ± 0.05	N.D.	0.04 ± 0.01	0.10 ± 0.05
Ellagic acid	29.67 ± 0.85	3.41 ± 0.01	9.39 ± 1.33	16.82 ± 3.28
Quercetin	1.41 ± 0.52	5.41 ± 0.19	0.30 ± 0.11	1.07 ± 0.47
Gallic acid	N.D.	N.D.	0.88 ± 0.11	2.02 ± 0.79
Chlorogenic acid	N.D.	N.D.	N.D.	N.D.
Caffeic acid	N.D.	N.D.	N.D.	N.D.
Syringic acid	0.06 ± 0.01	N.D.	0.01 ± 0.00	N.D.
Ferulic acid	N.D.	0.07 ± 0.02	0.06 ± 0.00	0.09 ± 0.06
p-Coumaric acid	N.D.	0.11 ± 0.01	N.D.	N.D.
Vanillic acid	0.20 ± 0.02	0.47 ± 0.02	N.D.	N.D.

Data are shown as the mean ± standard error (*n* = 3). N.D., not detected.

**Table 3:** The effect of various fruit powders on the content of PAHs in charcoal-grilled pork after different treatments.

Usage of Fruit Powder	Fruit Powder	Content of PAHs										Inhibition (%)	
		BaA		CHR		BbF		BaP		PAH4 *			
Meat Part		Belly	Loin	Belly	Loin	Belly	Loin	Belly	Loin	Belly	Loin	Belly	Loin
Spray	Control	28.47 ± 0.74 ^a^	1.28 ± 0.07 ^a^	17.55 ± 0.44 ^a^	0.62 ± 0.02 ^a^	13.13 ± 0.45 ^a^	N.D.	28.13 ± 0.33 ^a^	1.10 ± 0.01 ^a^	87.28 ± 1.96 ^a^	3.00 ± 0.49 ^a^	-	
	Guava	10.07 ± 0.35 ^d^	1.19 ± 0.03 ^ab^	6.10 ± 0.38 ^d^	0.53 ± 0.09 ^a^	4.24 ± 0.03 ^d^	N.D.	9.56 ± 0.12 ^c^	0.84 ± 0.12 ^b^	29.97 ± 0.87 ^d^	2.57 ± 0.25 ^ab^	65.7 ± 1.00 ^C^	14.3 ± 8.33 ^A^
	Lemon	13.24 ± 2.16 ^c^	0.75 ± 0.11 ^b^	8.10 ± 1.30 ^c^	0.40 ± 0.03 ^a^	5.46 ± 1.10 ^cd^	N.D.	11.70 ± 2.03 ^c^	0.95 ± 0.04 ^ab^	38.50 ± 6.60 ^c^	2.10 ± 0.18 ^b^	55.9 ± 7.56 ^BC^	30.0 ± 6.00 ^AB^
	Papaya	13.60 ± 0.22 ^c^	1.27 ± 0.08 ^a^	9.37 ± 0.15 ^c^	0.45 ± 0.09 ^a^	6.87 ± 0.12 ^c^	N.D.	15.20 ± 0.25 ^b^	0.89 ± 0.03 ^b^	45.04 ± 0.74 ^c^	2.62 ± 0.20 ^ab^	48.4 ± 0.85 ^B^	12.7 ± 6.67 ^A^
	Mango	24.85 ± 0.21 ^b^	1.08 ± 0.09 ^ab^	15.72 ± 0.12 ^b^	0.40 ± 0.13 ^a^	10.95 ± 0.02 ^b^	N.D.	25.74 ± 0.26 ^a^	0.82 ± 0.05 ^b^	77.26 ± 0.62 ^b^	2.31 ± 0.27 ^ab^	11.5 ± 0.71 ^A^	23.0 ± 9.00 ^A^
Marinade	Control	16.02 ± 0.46 ^a^	8.14 ± 0.12 ^a^	9.01 ± 0.23 ^a^	4.78 ± 0.12 ^a^	6.03 ± 0.17	2.06 ± 0.06 ^a^	12.67 ± 0.29 ^a^	3.98 ± 0.31 ^a^	43.74 ± 1.15 ^a^	18.96 ± 0.60 ^a^	-	-
	Guava	0.98 ± 0.01 ^d^	4.52 ± 0.08 ^b^	0.58 ± 0.09 ^b^	2.35 ± 0.05 ^c^	N.D.	0.50 ± 0.06 ^b^	0.77 ± 0.05 ^b^	2.18 ± 0.18 ^b^	2.33 ± 0.14 ^c^	9.55 ± 0.38 ^bc^	94.8 ± 0.32 ^E^	49.6 ± 2.00 ^B^
	Lemon	2.28 ± 0.09 ^c^	4.30 ± 0.01 ^b^	0.59 ± 0.29 ^b^	2.60 ± 0.06 ^b^	N.D.	0.61 ± 0.05 ^b^	1.01 ± 0.02 ^b^	2.39 ± 0.04 ^b^	3.88 ± 0.41 ^b^	9.91 ± 0.16 ^b^	91.1 ± 0.94 ^E^	47.7 ± 0.84 ^B^
	Papaya	2.56 ± 0.15 ^bc^	3.31 ± 0.14 ^c^	0.89 ± 0.18 ^b^	1.98 ± 0.10 ^d^	N.D.	0.32 ± 0.04 ^c^	1.01 ± 0.04 ^b^	1.35 ± 0.04 ^c^	4.46 ± 0.37 ^b^	6.96 ± 0.31 ^d^	89.8 ± 0.85 ^DE^	63.3 ± 1.64 ^BC^
	Mango	3.03 ± 0.04 ^b^	4.31 ± 0.06 ^b^	0.72 ± 0.03 ^b^	2.35 ± 0.04 ^c^	N.D.	0.32 ± 0.01 ^c^	1.06 ± 0.05 ^b^	1.73 ± 0.01 ^c^	4.81 ± 0.12 ^b^	8.71 ± 0.12 ^c^	89.0 ± 0.27 ^DE^	54.1 ± 0.63 ^B^
Mix	Control	71.77 ± 1.88 ^a^	9.72 ± 2.75 ^a^	46.72 ± 1.43 ^a^	6.02 ± 0.62 ^a^	44.24 ± 1.37 ^a^	3.95 ± 0.48 ^a^	92.23 ± 2.88 ^a^	7.82 ± 0.20 ^a^	254.96 ± 7.57 ^a^	27.51 ± 4.04 ^a^	-	-
	Guava	9.37 ± 0.04 ^cd^	2.23 ± 0.24 ^b^	5.25 ± 0.01 ^d^	1.16 ± 0.01 ^b^	3.37 ± 0.18 ^c^	0.41 ± 0.19 ^b^	6.57 ± 0.09 ^c^	1.68 ± 0.04 ^b^	24.57 ± 0.33 ^d^	5.47 ± 0.48 ^b^	90.4 ± 0.13 ^E^	80.1 ± 1.74 ^C^
	Lemon	14.35 ± 0.72 ^c^	2.07 ± 0.06 ^b^	8.52 ± 0.43 ^c^	1.49 ± 0.15 ^b^	6.96 ± 0.37 ^b^	0.54 ± 0.04 ^b^	13.54 ± 0.95 ^b^	1.57 ± 0.11 ^bc^	43.37 ± 2.47 ^c^	5.67 ± 0.36 ^b^	83.0 ± 0.97 ^DE^	79.4 ± 1.31 ^C^
	Papaya	21.65 ± 1.31 ^b^	1.95 ± 0.07 ^b^	11.90 ± 0.68 ^b^	1.41 ± 0.06 ^b^	7.52 ± 0.48 ^b^	0.35 ± 0.04 ^b^	14.19 ± 0.87 ^b^	1.46 ± 0.08 ^bc^	55.26 ± 3.34 ^b^	5.16 ± 0.25 ^b^	78.3 ± 1.31 ^D^	81.2 ± 0.91 ^C^
	Mango	11.75 ± 0.71 ^cd^	1.43 ± 0.06 ^b^	6.46 ± 0.19 ^d^	1.13 ± 0.32 ^b^	4.19 ± 0.24 ^c^	0.30 ± 0.04 ^b^	7.76 ± 0.26 ^c^	1.31 ± 0.03 ^bc^	30.16 ± 1.40 ^d^	4.13 ± 0.46 ^b^	88.2 ± 0.55 ^DE^	85.0 ± 1.67 ^C^

* PAH4 refers to the sum of all PAHs, including BaA, CHR, BbF, and BaP. Results are presented as the mean ± standard error (*n* = 3). Means with different lowercase letters in the same experimental group are significantly different (*p* < 0.05). Means with different uppercase letters between experimental groups are significantly different (*p* < 0.05). N.D., not detected.

**Table 4:** Estimated daily intake (EDI) and margin of effect (MOE) of the general population.

Usage of Fruit Powder	Fruit Powder	BaP				PAH4			
Meat Type		Belly		Loin		Belly		Loin	
Indicator		EDI (ng/kg bw/day)	MOE	EDI (ng/kg bw/day)	MOE	EDI (ng/kg bw/day)	MOE	EDI (ng/kg bw/day)	MOE
Solution spray	Control	3.94	1.8 × 10^4^	0.04	1.6 × 10^6^	12.22	3.0 × 10^4^	0.12	2.8 × 10^6^
	Guava	1.34	5.2 × 10^4^	0.03	2.0 × 10^6^	4.20	8.1 × 10^4^	0.10	3.3 × 10^6^
	Lemon	1.64	4.3 × 10^4^	0.04	1.8 × 10^6^	5.40	6.3 × 10^4^	0.07	4.0 × 10^6^
	Papaya	2.13	3.3 × 10^4^	0.04	2.0 × 10^6^	6.31	5.4 × 10^4^	0.11	3.2 × 10^6^
	Mango	3.60	2.0 × 10^4^	0.03	2.1 × 10^6^	10.82	3.1 × 10^4^	0.09	3.7 × 10^6^
Marinade	Control	1.77	3.9 × 10^4^	0.16	4.4 × 10^5^	6.12	5.6 × 10^4^	0.76	4.5 × 10^5^
	Guava	0.11	6.5 × 10^4^	0.09	8.0 × 10^5^	0.47	7.2 × 10^5^	0.38	8.9 × 10^5^
	Lemon	0.14	5.0 × 10^4^	0.10	7.3 × 10^5^	0.54	6.3 × 10^5^	0.40	8.6 × 10^5^
	Papaya	0.14	5.0 × 10^4^	0.05	1.3 × 10^6^	0.62	5.4 × 10^5^	0.28	1.2 × 10^6^
	Mango	0.15	4.7 × 10^4^	0.07	1.0 × 10^6^	0.67	5.0 × 10^5^	0.35	9.8 × 10^5^
Mix	Control	12.91	5.4 × 10^3^	0.31	2.2 × 10^5^	35.70	9.5 × 10^3^	1.10	3.1 × 10^5^
	Guava	0.92	7.6 × 10^4^	0.07	1.0 × 10^6^	3.44	9.9 × 10^4^	0.22	1.6 × 10^6^
	Lemon	1.90	3.7 × 10^4^	0.06	1.1 × 10^6^	6.07	5.6 × 10^4^	0.23	1.5 × 10^6^
	Papaya	1.99	7.6 × 10^4^	0.06	1.2 × 10^6^	7.74	4.4 × 10^4^	0.21	1.6 × 10^6^
	Mango	1.09	6.4 × 10^4^	0.05	1.3 × 10^6^	4.22	8.1 × 10^4^	0.17	2.1 × 10^6^

**Table 5:** Triangle test (n = 10).

Panelist ID	Sample A	Sample B	Sample C	Odd Sample Identified (A, B, or C)	Correct (Yes/No)
1	Grilled with	Grilled without	Grilled without	C	No
2	Grilled with	Grilled without	Grilled without	A	Yes
3	Grilled with	Grilled with	Grilled without	B	No
4	Grilled without	Grilled with	Grilled without	A	No
5	Grilled with	Grilled without	Grilled with	A	No
6	Grilled without	Grilled without	Grilled with	B	No
7	Grilled without	Grilled with	Grilled without	B	Yes
8	Grilled without	Grilled with	Grilled without	C	No
9	Grilled with	Grilled without	Grilled with	A	No
10	Grilled without	Grilled with	Grilled without	C	No
**Description**					**Percentage of correct responses (%)**
Different					20% ^a^
Same					80% ^a^

Significance was assessed using the minimum number of correct responses as specified by ISO 4120:2016. Results with the same letter are considered non-significant (*p* > 0.05).

**Table 6:** Sensory evaluation (n = 10).

Indicator	Average Score		*p*-Value
	Control	Experimental	
Appearance	4.4	5.3	0.06
Smell	5.0	5.0	1.0
Flavor	4.5	4.6	0.85
Level of greasiness	4.5	4.4	0.89
Overall acceptance	4.6	4.9	0.46

All data were analyzed by SAS using a two-way analysis of variance (ANOVA), and significant difference between the control and experimental groups was identified at *p* < 0.05.

## Data Availability

The original contributions presented in the study are included in the article/Supplementary Material, further inquiries can be directed to the corresponding author.

## Supplementary Materials

The following supporting information can be downloaded at: https://www.mdpi.com/article/10.3390/foods13193179/s1.
