# Supplementary material for: Inhibitory Effects of Fruit Powders on the Formation of Polycyclic Aromatic Hydrocarbons in Charcoal-Grilled Pork
Source: Foods. 2024 Oct 6;13(19):3179. doi: 10.3390/foods13193179 (PMC11475276; doi:10.3390/foods13193179)
Supplement: Supplementary file 1 [file foods-13-03179-s001.zip › foods-3232258-supplementary.pdf]

Supplementary Material

Triangle Test Instructions

Overview:

In this test, you will evaluate three samples of a specific product. Among these samples, two are identical, and one is different. Your task is to taste the samples carefully and identify which one you believe differs from the other two. Please note that there is no right or wrong answer as we assess your perception of sensory differences.

Before the test begins, you will be provided with the following items to assist you during the evaluation process:

- ✓ A cup of plain water for palate cleansing.
- ✓ A spitting cup for discarding water or excess samples if needed.
- ✓ A tissue for any necessary cleanup.
- ✓ A pair of chopsticks to handle the samples.

If you notice any missing items or have any concerns or questions during the test, please raise your hand to alert the staff for immediate assistance.

Instructions for the test:

1. **Palate Cleansing (Pre-test Preparation):**
   Before tasting, cleanse your palate by rinsing your mouth with plain water for 30 seconds. This step is crucial to remove any residual flavors or aftertaste that might interfere with your ability to detect differences among the samples. After rinsing, spit out the water into the spitting cup provided. Repeat this process if needed to ensure a neutral palate before starting.
2. **Evaluation Process (Tasting):**
   In front of you, there are three samples labeled with random codes. Taste the samples one by one, moving from left to right. Pay close attention to their flavor, texture, and other sensory attributes. Your goal is to identify the sample that differs from the other two.
   - **First Sample:** Take a small portion using the chopsticks provided. Taste it carefully, focusing on the sensory characteristics (flavor, texture, aroma).
   - **Second Sample:** After cleansing your palate, move on to the second sample and repeat the same steps.
   - **Third Sample:** Cleanse your palate again and taste the final sample.
3. **Palate Reset (Between Samples):**
   After tasting each sample, rinse your mouth with water for at least 15-30 seconds to cleanse your palate before proceeding to the following sample. This is essential to ensure that the flavors of the previous sample do not influence each tasting. Be sure to spit out the water after rinsing.
4. **Decision Making (Identifying the Odd Sample):**

Once you have tasted all three samples, decide which one is distinct from the other two. Remember that the test is not about preference but identifying the sample that stands out regarding sensory differences. Take your time to compare the samples before making your final decision.

5. **Recording Your Response:**
   Once chosen, mark the sample code you believe is different on the answer sheet provided. Please ensure your response is clear and accurate before submitting your answer to the test administrator.

Response after testing:

**Panelist ID**	**Sample A**	**Sample B**	**Sample C**	**Odd Sample Identified (A, B, or C)**

Acceptance Test Instructions

(7-Point Scale Scoring Difference Test)

■ Experiment Procedure:

1. **Sample Presentation:**
   - Each sample will be presented to you one at a time. Please take your time to evaluate the sample carefully.
   - Once you have completed evaluating a sample, record your responses on the score sheet provided. All questions about each sample must be fully answered before proceeding to the next.
2. **Sample Collection:**
   - After you finish a sample, the testing staff will collect it before serving the next one.
   - If you need additional time to complete your evaluation, please inform the staff to avoid rushing.
3. **Test Setup and Materials:**
   - You will be provided with the following items:
     (1) A cup of water to rinse your mouth.
     (2) A spitting cup to dispose of water or any residual sample if necessary.
     (3) A tissue for any cleanup or wiping needs.
     (4) A pair of chopsticks to handle the sample.
   - If any of the items are missing or if you encounter any issues during the test, please raise your hand, and a staff member will assist you promptly.

■ Scoring Method:

1. **Preparation Before Tasting:**
   - Before tasting each sample, start by rinsing your mouth thoroughly with the water provided for **30 seconds** to cleanse your palate. This step is crucial to eliminate lingering flavors from previous samples and ensure an unbiased evaluation of the next one.
2. **Sensory Evaluation Process:**
   - **Visual Inspection**: Open the sample container and first take a moment to observe the appearance of the sample. Note any distinguishing features such as color, texture, or overall presentation.
   - **Aroma Assessment:** After visually inspecting the sample, gently smell it to assess the aroma. This step helps capture the initial sensory impression of the sample before tasting.
   - **Taste the Sample:** Proceed to taste the sample using the chopsticks provided. Focus on the flavor, texture, and other sensory attributes relevant to the evaluation.
   - **Recording Your Preference:**

(1) Based on your overall impression (appearance, aroma, taste, and texture), indicate your preference by marking a "✓" in the corresponding box on the 7-point scale provided on your score sheet.

(2) The scale ranges from 1 (extremely dislike) to 7 (extremely like). Be sure to carefully reflect on your overall experience with each sample before marking your response.

3. **Resting Between Samples**:
   - After you have finished scoring one sample, take the time to **rinse your mouth again for 30 seconds** before the following sample is presented.
   - It is recommended that you **rest for 2-3 minutes** between samples to allow your palate to fully recover, ensuring that your evaluation of the subsequent sample is as objective as possible.

**Panelist ID: ____________**<br>**Sample ID: ____________**	**Appearance**	**Smell**	**Texture**	**Flavor**	**Greasy level**	**Overall acceptance**
1. Like extremely	☐	☐	☐	☐	☐	☐
2. Like moderately	☐	☐	☐	☐	☐	☐
3. Like slightly	☐	☐	☐	☐	☐	☐
4. Neither like nor dislike	☐	☐	☐	☐	☐	☐
5. Dislike slightly	☐	☐	☐	☐	☐	☐
6. Dislike moderately	☐	☐	☐	☐	☐	☐
7. Dislike extremely	☐	☐	☐	☐	☐	☐

Sensory Evaluation Participant Consent Form

Consent Form Number: 1

- **Research Title:**
  Inhibition of Polycyclic Aromatic Hydrocarbons Formation in Charcoal-Grilled Pork Using Phenolic-Rich Fruit Powders
- **Principal Investigator:**
  Professor Kuo-Chiang Hsu
- **Research Institution:**
  Department of Nutrition, China Medical University, Taiwan
- **Purpose of the Study:**
  This study aimed to investigate the feasibility of inhibiting the formation of four kinds of PAHs (BaA, CHR, BbF, and BaP) in charcoal-grilled pork belly and loin by spraying, marinating, and mixing with four freeze-dried fruit powders (lemon, guava, papaya, and mango). For sensory evaluation, the assessors will be asked to score the meat according to their preferences regarding its appearance, smell, texture, flavor, greasy level, and overall acceptance through sensory evaluation methods. Your participation will help us better understand and analyze the sensory quality of food to improve future food research and development.
- **Participant Requirements:**
  - [x] You must be at least 18 years old.
  - [x] You must not have any food allergies or food-related health issues.
  - [x] You must agree to participate in multiple evaluation sessions as required by the study.
- **Study Procedures:**
  You will be asked to taste a certain number of food items and evaluate their sensory characteristics. The entire evaluation process is expected to take approximately 15 minutes. All evaluation results will be processed anonymously and used solely for data analysis in this study.
- **Risks and Benefits:**
  Participation in this study may involve tasting different types of food, which could cause minor discomfort (such as slight oral irritation). However, all provided foods will meet food safety standards. Participation in this study will not provide any financial benefits, but your contribution will be valuable to food research.
- **Confidentiality:**
  All your personal information and evaluation results will be kept strictly confidential and only accessible to the research team. Study data will be reported anonymously and will not be traceable to any individual.

- **Voluntary Participation:**

  Your participation is entirely voluntary, and you have the right to withdraw from the study at any time without providing a reason, and without facing any penalty or adverse consequences.

- **Contact Information:**

  If you have any questions or concerns, you may contact the principal investigator at any time.

  Phone: 0918-268-020

  Email: jane.belle.bj@gmail.com

- **Participant Statement:**

  I have read and understood the above information and agree to participate in this study. I understand my rights and the potential risks, and I agree to follow the guidelines for sensory evaluation during the study.

Participant Signature:

Yun-Hsuan Chen Date: 2023 / 8 / 16

Researcher Signature:

Kuo Chiang Hsu Date: 2023 / 8 / 16

Sensory Evaluation Participant Consent Form

Consent Form Number: 2

- **Research Title:**
  Inhibition of Polycyclic Aromatic Hydrocarbons Formation in Charcoal-Grilled Pork Using Phenolic-Rich Fruit Powders
- **Principal Investigator:**
  Professor Kuo-Chiang Hsu
- **Research Institution:**
  Department of Nutrition, China Medical University, Taiwan
- **Purpose of the Study:**
  This study aimed to investigate the feasibility of inhibiting the formation of four kinds of PAHs (BaA, CHR, BbF, and BaP) in charcoal-grilled pork belly and loin by spraying, marinating, and mixing with four freeze-dried fruit powders (lemon, guava, papaya, and mango). For sensory evaluation, the assessors will be asked to score the meat according to their preferences regarding its appearance, smell, texture, flavor, greasy level, and overall acceptance through sensory evaluation methods. Your participation will help us better understand and analyze the sensory quality of food to improve future food research and development.
- **Participant Requirements:**

  ☑ You must be at least 18 years old.

  ☑ You must not have any food allergies or food-related health issues.

  ☑ You must agree to participate in multiple evaluation sessions as required by the study.

- **Study Procedures:**
  You will be asked to taste a certain number of food items and evaluate their sensory characteristics. The entire evaluation process is expected to take approximately 15 minutes. All evaluation results will be processed anonymously and used solely for data analysis in this study.
- **Risks and Benefits:**
  Participation in this study may involve tasting different types of food, which could cause minor discomfort (such as slight oral irritation). However, all provided foods will meet food safety standards. Participation in this study will not provide any financial benefits, but your contribution will be valuable to food research.
- **Confidentiality:**
  All your personal information and evaluation results will be kept strictly confidential and only accessible to the research team. Study data will be reported anonymously and will not be traceable to any individual.

- **Voluntary Participation:**

  Your participation is entirely voluntary, and you have the right to withdraw from the study at any time without providing a reason, and without facing any penalty or adverse consequences.

- **Contact Information:**

  If you have any questions or concerns, you may contact the principal investigator at any time.

  Phone: 0905419899

  Email: rita910625@gmail.com

- **Participant Statement:**

  I have read and understood the above information and agree to participate in this study. I understand my rights and the potential risks, and I agree to follow the guidelines for sensory evaluation during the study.

Participant Signature:

Rita Yang. Date: 2023 / 8 / 16.

Researcher Signature:

Kuo Chiang Hsu Date: 2023 / 8 / 16

Sensory Evaluation Participant Consent Form

Consent Form Number: 3

- **Research Title:**
  Inhibition of Polycyclic Aromatic Hydrocarbons Formation in Charcoal-Grilled Pork Using Phenolic-Rich Fruit Powders
- **Principal Investigator:**
  Professor Kuo-Chiang Hsu
- **Research Institution:**
  Department of Nutrition, China Medical University, Taiwan
- **Purpose of the Study:**
  This study aimed to investigate the feasibility of inhibiting the formation of four kinds of PAHs (BaA, CHR, BbF, and BaP) in charcoal-grilled pork belly and loin by spraying, marinating, and mixing with four freeze-dried fruit powders (lemon, guava, papaya, and mango). For sensory evaluation, the assessors will be asked to score the meat according to their preferences regarding its appearance, smell, texture, flavor, greasy level, and overall acceptance through sensory evaluation methods. Your participation will help us better understand and analyze the sensory quality of food to improve future food research and development.
- **Participant Requirements:**
  - [x] You must be at least 18 years old.
  - [x] You must not have any food allergies or food-related health issues.
  - [x] You must agree to participate in multiple evaluation sessions as required by the study.
- **Study Procedures:**
  You will be asked to taste a certain number of food items and evaluate their sensory characteristics. The entire evaluation process is expected to take approximately 15 minutes. All evaluation results will be processed anonymously and used solely for data analysis in this study.
- **Risks and Benefits:**
  Participation in this study may involve tasting different types of food, which could cause minor discomfort (such as slight oral irritation). However, all provided foods will meet food safety standards. Participation in this study will not provide any financial benefits, but your contribution will be valuable to food research.
- **Confidentiality:**
  All your personal information and evaluation results will be kept strictly confidential and only accessible to the research team. Study data will be reported anonymously and will not be traceable to any individual.

- **Voluntary Participation:**

  Your participation is entirely voluntary, and you have the right to withdraw from the study at any time without providing a reason, and without facing any penalty or adverse consequences.

- **Contact Information:**

  If you have any questions or concerns, you may contact the principal investigator at any time.

  Phone: 0978-706-990

  Email: carol510020@gmail.com

- **Participant Statement:**

  I have read and understood the above information and agree to participate in this study. I understand my rights and the potential risks, and I agree to follow the guidelines for sensory evaluation during the study.

Participant Signature:

Yung Hui Chung Date: 2023 / 8 / 16

Researcher Signature:

Kuo Chiang Hsu Date: 2023 / 8 / 16

Sensory Evaluation Participant Consent Form

Consent Form Number: 4

- **Research Title:**
  Inhibition of Polycyclic Aromatic Hydrocarbons Formation in Charcoal-Grilled Pork Using Phenolic-Rich Fruit Powders
- **Principal Investigator:**
  Professor Kuo-Chiang Hsu
- **Research Institution:**
  Department of Nutrition, China Medical University, Taiwan
- **Purpose of the Study:**
  This study aimed to investigate the feasibility of inhibiting the formation of four kinds of PAHs (BaA, CHR, BbF, and BaP) in charcoal-grilled pork belly and loin by spraying, marinating, and mixing with four freeze-dried fruit powders (lemon, guava, papaya, and mango). For sensory evaluation, the assessors will be asked to score the meat according to their preferences regarding its appearance, smell, texture, flavor, greasy level, and overall acceptance through sensory evaluation methods. Your participation will help us better understand and analyze the sensory quality of food to improve future food research and development.
- **Participant Requirements:**

  ☑ You must be at least 18 years old.

  ☑ You must not have any food allergies or food-related health issues.

  ☑ You must agree to participate in multiple evaluation sessions as required by the study.

- **Study Procedures:**
  You will be asked to taste a certain number of food items and evaluate their sensory characteristics. The entire evaluation process is expected to take approximately 15 minutes. All evaluation results will be processed anonymously and used solely for data analysis in this study.
- **Risks and Benefits:**
  Participation in this study may involve tasting different types of food, which could cause minor discomfort (such as slight oral irritation). However, all provided foods will meet food safety standards. Participation in this study will not provide any financial benefits, but your contribution will be valuable to food research.
- **Confidentiality:**
  All your personal information and evaluation results will be kept strictly confidential and only accessible to the research team. Study data will be reported anonymously and will not be traceable to any individual.

- **Voluntary Participation:**

  Your participation is entirely voluntary, and you have the right to withdraw from the study at any time without providing a reason, and without facing any penalty or adverse consequences.

- **Contact Information:**

  If you have any questions or concerns, you may contact the principal investigator at any time.

  Phone: +886 988793051

  Email: shihann98@gmail.com

- **Participant Statement:**

  I have read and understood the above information and agree to participate in this study. I understand my rights and the potential risks, and I agree to follow the guidelines for sensory evaluation during the study.

Participant Signature:

SHIH EN RUAN Date: 2023 / 8 / 16

Researcher Signature:

Kuo Chiang Hsu Date: 2023 / 8 / 16

Sensory Evaluation Participant Consent Form

Consent Form Number: 5

- **Research Title:**
  Inhibition of Polycyclic Aromatic Hydrocarbons Formation in Charcoal-Grilled Pork Using Phenolic-Rich Fruit Powders
- **Principal Investigator:**
  Professor Kuo-Chiang Hsu
- **Research Institution:**
  Department of Nutrition, China Medical University, Taiwan
- **Purpose of the Study:**
  This study aimed to investigate the feasibility of inhibiting the formation of four kinds of PAHs (BaA, CHR, BbF, and BaP) in charcoal-grilled pork belly and loin by spraying, marinating, and mixing with four freeze-dried fruit powders (lemon, guava, papaya, and mango). For sensory evaluation, the assessors will be asked to score the meat according to their preferences regarding its appearance, smell, texture, flavor, greasy level, and overall acceptance through sensory evaluation methods. Your participation will help us better understand and analyze the sensory quality of food to improve future food research and development.
- **Participant Requirements:**

  ☑ You must be at least 18 years old.

  ☑ You must not have any food allergies or food-related health issues.

  ☑ You must agree to participate in multiple evaluation sessions as required by the study.

- **Study Procedures:**
  You will be asked to taste a certain number of food items and evaluate their sensory characteristics. The entire evaluation process is expected to take approximately 15 minutes. All evaluation results will be processed anonymously and used solely for data analysis in this study.
- **Risks and Benefits:**
  Participation in this study may involve tasting different types of food, which could cause minor discomfort (such as slight oral irritation). However, all provided foods will meet food safety standards. Participation in this study will not provide any financial benefits, but your contribution will be valuable to food research.
- **Confidentiality:**
  All your personal information and evaluation results will be kept strictly confidential and only accessible to the research team. Study data will be reported anonymously and will not be traceable to any individual.

- **Voluntary Participation:**

  Your participation is entirely voluntary, and you have the right to withdraw from the study at any time without providing a reason, and without facing any penalty or adverse consequences.

- **Contact Information:**

  If you have any questions or concerns, you may contact the principal investigator at any time.

  Phone: 0921786764

  Email: u112076006@cmu.edu.tw

- **Participant Statement:**

  I have read and understood the above information and agree to participate in this study. I understand my rights and the potential risks, and I agree to follow the guidelines for sensory evaluation during the study.

Participant Signature:

Bo-Syuan Su Date: 2023 / 08 / 16

Researcher Signature:

Kuo Chiang Hsu Date: 2023 / 8 / 16

Sensory Evaluation Participant Consent Form

Consent Form Number: 6

- **Research Title:**
  Inhibition of Polycyclic Aromatic Hydrocarbons Formation in Charcoal-Grilled Pork Using Phenolic-Rich Fruit Powders
- **Principal Investigator:**
  Professor Kuo-Chiang Hsu
- **Research Institution:**
  Department of Nutrition, China Medical University, Taiwan
- **Purpose of the Study:**
  This study aimed to investigate the feasibility of inhibiting the formation of four kinds of PAHs (BaA, CHR, BbF, and BaP) in charcoal-grilled pork belly and loin by spraying, marinating, and mixing with four freeze-dried fruit powders (lemon, guava, papaya, and mango). For sensory evaluation, the assessors will be asked to score the meat according to their preferences regarding its appearance, smell, texture, flavor, greasy level, and overall acceptance through sensory evaluation methods. Your participation will help us better understand and analyze the sensory quality of food to improve future food research and development.
- **Participant Requirements:**

  ☑ You must be at least 18 years old.

  ☑ You must not have any food allergies or food-related health issues.

  ☑ You must agree to participate in multiple evaluation sessions as required by the study.
- **Study Procedures:**
  You will be asked to taste a certain number of food items and evaluate their sensory characteristics. The entire evaluation process is expected to take approximately 15 minutes. All evaluation results will be processed anonymously and used solely for data analysis in this study.
- **Risks and Benefits:**
  Participation in this study may involve tasting different types of food, which could cause minor discomfort (such as slight oral irritation). However, all provided foods will meet food safety standards. Participation in this study will not provide any financial benefits, but your contribution will be valuable to food research.
- **Confidentiality:**
  All your personal information and evaluation results will be kept strictly confidential and only accessible to the research team. Study data will be reported anonymously and will not be traceable to any individual.

- **Voluntary Participation:**

  Your participation is entirely voluntary, and you have the right to withdraw from the study at any time without providing a reason, and without facing any penalty or adverse consequences.

- **Contact Information:**

  If you have any questions or concerns, you may contact the principal investigator at any time.

  Phone: 0935817474

  Email: yoyo09300524@gmail.com.tw

- **Participant Statement:**

  I have read and understood the above information and agree to participate in this study. I understand my rights and the potential risks, and I agree to follow the guidelines for sensory evaluation during the study.

Participant Signature:

Juan Huang Date: 2023/ 08 / 16

Researcher Signature:

Kuo Chang Hsu Date: 2023/ 8 / 16

Sensory Evaluation Participant Consent Form

Consent Form Number: 7

- **Research Title:**
  Inhibition of Polycyclic Aromatic Hydrocarbons Formation in Charcoal-Grilled Pork Using Phenolic-Rich Fruit Powders
- **Principal Investigator:**
  Professor Kuo-Chiang Hsu
- **Research Institution:**
  Department of Nutrition, China Medical University, Taiwan
- **Purpose of the Study:**
  This study aimed to investigate the feasibility of inhibiting the formation of four kinds of PAHs (BaA, CHR, BbF, and BaP) in charcoal-grilled pork belly and loin by spraying, marinating, and mixing with four freeze-dried fruit powders (lemon, guava, papaya, and mango). For sensory evaluation, the assessors will be asked to score the meat according to their preferences regarding its appearance, smell, texture, flavor, greasy level, and overall acceptance through sensory evaluation methods. Your participation will help us better understand and analyze the sensory quality of food to improve future food research and development.
- **Participant Requirements:**

  ☑ You must be at least 18 years old.

  ☑ You must not have any food allergies or food-related health issues.

  ☑ You must agree to participate in multiple evaluation sessions as required by the study.
- **Study Procedures:**
  You will be asked to taste a certain number of food items and evaluate their sensory characteristics. The entire evaluation process is expected to take approximately 15 minutes. All evaluation results will be processed anonymously and used solely for data analysis in this study.
- **Risks and Benefits:**
  Participation in this study may involve tasting different types of food, which could cause minor discomfort (such as slight oral irritation). However, all provided foods will meet food safety standards. Participation in this study will not provide any financial benefits, but your contribution will be valuable to food research.
- **Confidentiality:**
  All your personal information and evaluation results will be kept strictly confidential and only accessible to the research team. Study data will be reported anonymously and will not be traceable to any individual.

- **Voluntary Participation:**

  Your participation is entirely voluntary, and you have the right to withdraw from the study at any time without providing a reason, and without facing any penalty or adverse consequences.

- **Contact Information:**

  If you have any questions or concerns, you may contact the principal investigator at any time.

  Phone: 0963 783 325

  Email: kitty54001@gmail.com

- **Participant Statement:**

  I have read and understood the above information and agree to participate in this study. I understand my rights and the potential risks, and I agree to follow the guidelines for sensory evaluation during the study.

Participant Signature:

Lin-Xiao-Wen Date: 2023/ 8 / 16

Researcher Signature:

Kuo Chiang Hsu Date: 2023 8 / 16

Sensory Evaluation Participant Consent Form

Consent Form Number: 8

- **Research Title:**
  Inhibition of Polycyclic Aromatic Hydrocarbons Formation in Charcoal-Grilled Pork Using Phenolic-Rich Fruit Powders
- **Principal Investigator:**
  Professor Kuo-Chiang Hsu
- **Research Institution:**
  Department of Nutrition, China Medical University, Taiwan
- **Purpose of the Study:**
  This study aimed to investigate the feasibility of inhibiting the formation of four kinds of PAHs (BaA, CHR, BbF, and BaP) in charcoal-grilled pork belly and loin by spraying, marinating, and mixing with four freeze-dried fruit powders (lemon, guava, papaya, and mango). For sensory evaluation, the assessors will be asked to score the meat according to their preferences regarding its appearance, smell, texture, flavor, greasy level, and overall acceptance through sensory evaluation methods. Your participation will help us better understand and analyze the sensory quality of food to improve future food research and development.
- **Participant Requirements:**

  ☑ You must be at least 18 years old.

  ☑ You must not have any food allergies or food-related health issues.

  ☑ You must agree to participate in multiple evaluation sessions as required by the study.

- **Study Procedures:**
  You will be asked to taste a certain number of food items and evaluate their sensory characteristics. The entire evaluation process is expected to take approximately 15 minutes. All evaluation results will be processed anonymously and used solely for data analysis in this study.
- **Risks and Benefits:**
  Participation in this study may involve tasting different types of food, which could cause minor discomfort (such as slight oral irritation). However, all provided foods will meet food safety standards. Participation in this study will not provide any financial benefits, but your contribution will be valuable to food research.
- **Confidentiality:**
  All your personal information and evaluation results will be kept strictly confidential and only accessible to the research team. Study data will be reported anonymously and will not be traceable to any individual.

- **Voluntary Participation:**

  Your participation is entirely voluntary, and you have the right to withdraw from the study at any time without providing a reason, and without facing any penalty or adverse consequences.

- **Contact Information:**

  If you have any questions or concerns, you may contact the principal investigator at any time.

  Phone: 0908807364

  Email: kimcmu113@gmail.com

- **Participant Statement:**

  I have read and understood the above information and agree to participate in this study. I understand my rights and the potential risks, and I agree to follow the guidelines for sensory evaluation during the study.

Participant Signature:

Yi-chieh-Hsieh Date: 2023/ 8 / 16

Researcher Signature:

Kuo Chiang Hsu Date: 2023/ 8 / 16

Sensory Evaluation Participant Consent Form

Consent Form Number: 9

- **Research Title:**
  Inhibition of Polycyclic Aromatic Hydrocarbons Formation in Charcoal-Grilled Pork Using Phenolic-Rich Fruit Powders
- **Principal Investigator:**
  Professor Kuo-Chiang Hsu
- **Research Institution:**
  Department of Nutrition, China Medical University, Taiwan
- **Purpose of the Study:**
  This study aimed to investigate the feasibility of inhibiting the formation of four kinds of PAHs (BaA, CHR, BbF, and BaP) in charcoal-grilled pork belly and loin by spraying, marinating, and mixing with four freeze-dried fruit powders (lemon, guava, papaya, and mango). For sensory evaluation, the assessors will be asked to score the meat according to their preferences regarding its appearance, smell, texture, flavor, greasy level, and overall acceptance through sensory evaluation methods. Your participation will help us better understand and analyze the sensory quality of food to improve future food research and development.
- **Participant Requirements:**

  ☑ You must be at least 18 years old.

  ☑ You must not have any food allergies or food-related health issues.

  ☑ You must agree to participate in multiple evaluation sessions as required by the study.

- **Study Procedures:**
  You will be asked to taste a certain number of food items and evaluate their sensory characteristics. The entire evaluation process is expected to take approximately 15 minutes. All evaluation results will be processed anonymously and used solely for data analysis in this study.
- **Risks and Benefits:**
  Participation in this study may involve tasting different types of food, which could cause minor discomfort (such as slight oral irritation). However, all provided foods will meet food safety standards. Participation in this study will not provide any financial benefits, but your contribution will be valuable to food research.
- **Confidentiality:**
  All your personal information and evaluation results will be kept strictly confidential and only accessible to the research team. Study data will be reported anonymously and will not be traceable to any individual.

- **Voluntary Participation:**

  Your participation is entirely voluntary, and you have the right to withdraw from the study at any time without providing a reason, and without facing any penalty or adverse consequences.

- **Contact Information:**

  If you have any questions or concerns, you may contact the principal investigator at any time.

  Phone: 0965497968

  Email: f916110@gmail.com

- **Participant Statement:**

  I have read and understood the above information and agree to participate in this study. I understand my rights and the potential risks, and I agree to follow the guidelines for sensory evaluation during the study.

Participant Signature:

CHIA YU LIN Date: 2023 / 8 / 16

Researcher Signature:

Kuo Chiang Hsu Date: 2023 / 8 / 16

Sensory Evaluation Participant Consent Form

Consent Form Number: 10

- **Research Title:**
  Inhibition of Polycyclic Aromatic Hydrocarbons Formation in Charcoal-Grilled Pork Using Phenolic-Rich Fruit Powders
- **Principal Investigator:**
  Professor Kuo-Chiang Hsu
- **Research Institution:**
  Department of Nutrition, China Medical University, Taiwan
- **Purpose of the Study:**
  This study aimed to investigate the feasibility of inhibiting the formation of four kinds of PAHs (BaA, CHR, BbF, and BaP) in charcoal-grilled pork belly and loin by spraying, marinating, and mixing with four freeze-dried fruit powders (lemon, guava, papaya, and mango). For sensory evaluation, the assessors will be asked to score the meat according to their preferences regarding its appearance, smell, texture, flavor, greasy level, and overall acceptance through sensory evaluation methods. Your participation will help us better understand and analyze the sensory quality of food to improve future food research and development.
- **Participant Requirements:**

  ☑ You must be at least 18 years old.

  ☑ You must not have any food allergies or food-related health issues.

  ☑ You must agree to participate in multiple evaluation sessions as required by the study.

- **Study Procedures:**
  You will be asked to taste a certain number of food items and evaluate their sensory characteristics. The entire evaluation process is expected to take approximately 15 minutes. All evaluation results will be processed anonymously and used solely for data analysis in this study.
- **Risks and Benefits:**
  Participation in this study may involve tasting different types of food, which could cause minor discomfort (such as slight oral irritation). However, all provided foods will meet food safety standards. Participation in this study will not provide any financial benefits, but your contribution will be valuable to food research.
- **Confidentiality:**
  All your personal information and evaluation results will be kept strictly confidential and only accessible to the research team. Study data will be reported anonymously and will not be traceable to any individual.

- **Voluntary Participation:**

  Your participation is entirely voluntary, and you have the right to withdraw from the study at any time without providing a reason, and without facing any penalty or adverse consequences.

- **Contact Information:**

  If you have any questions or concerns, you may contact the principal investigator at any time.

  Phone: 0958125101

  Email: yijin16@gmail.com

- **Participant Statement:**

  I have read and understood the above information and agree to participate in this study. I understand my rights and the potential risks, and I agree to follow the guidelines for sensory evaluation during the study.

Participant Signature:

Rhea Wu Date: 2023/ 8 / 16

Researcher Signature:

Kuo Chiang Hsu Date: 2023/ 8 / 16
